# Investigation of the optimum planting dates for maize varieties using a hybrid approach: A case of Hwedza, Zimbabwe

**DOI:** 10.1016/j.heliyon.2021.e06109

**Published:** 2021-02-06

**Authors:** Hillary Mugiyo, Teddious Mhizha, Vimbayi.G.P. Chimonyo, Tafadzwanashe Mabhaudhi

**Affiliations:** a Centre for Transformative Agricultural and Food Systems, School of Agricultural, Earth and Environmental Sciences, University of KwaZulu-Natal, P. Bag X01, Scottsville, Pietermaritzburg, 3209, South Africa; b Department of Physics, Faculty of Science, University of Zimbabwe, 630 Churchill Avenue, Harare, Zimbabwe

**Keywords:** Crop modelling, DSSAT, Climate variability, Maize varieties, Cropping calendar

## Abstract

Water scarcity and unreliable weather conditions frequently cause smallholder farmers in Zimbabwe to plant maize (*Zea mays L.)* varieties outside the optimum planting timeframe. This challenge exacts the necessity to develop sowing management options for decision support. The study's objective was to use a hybrid approach to determine the best planting windows and maize varieties. The combination will guide farmers on planting dates, dry spell probability during critical stages of the crop growth cycle and rainfall cessation. To capture farmer's perception on agroclimatic information, a systematic random sampling of 438 smallholders was carried out. An analysis of climatic data during 1949–2012 was conducted using INSTAT to identify the best planting criterion. The best combination of planting criterion and maize varieties analysis was then achieved by optimizing planting dates and maize varieties in the DSSAT environment. It was found that 56.2% of farmers grew short-season varieties, 40.2% medium-season varieties and 3.6% long-season varieties. It was also established that the number of rain days and maize yield had a strong positive relationship (p = 0.0049). No significant association was found amongst maize yield (p > 0.05), and planting date criteria, Depth (40mm in 4 days), the AREX criterion- Agricultural Research Extension (25 mm rainfall in 7 days) and the MET Criterion-Department of Meteorological Services (40 mm in 15 days). Highest yields were simulated under the combination of medium-season maize variety and the AREX and MET criteria. The range of simulated yields from 0.0 t/ha to 2.8 t/ha formed the basis for the development of an operational decision support tool (cropping calendar) with (RMSE) (0.20). The methodology can be used to select the best suitable maize varieties and a range of planting time.

## 1 Introduction

In rural sub-Saharan Africa (SSA) and resource-constrained smallholder, rain-fed agriculture is the most important sector for providing food security (Gowing and Palmer, 2008). Rain-fed crop production is becoming more unreliable, yet 90% maize farmers of rural farming communities relay of rain-fed agriculture as a livelihood strategy (Kirkegaard et al., 2014; Sheffield et al., 2014). Maize production in Zimbabwe decreased dramatically over the past decade, yet over 50 percent of maize human consumption is imported from African, America, Asian and European countries (FNSWG, 2015). The production of maize in Zimbabwe varies from 950 000 tonnes (1 500 000 ha) to 2 500 000 tonnes (2 000 000 ha) per annum (OECD and FAO, 2016). In Zimbabwe, maize is a strategic crop, therefore improving its production through good agricultural practices, especially in marginal areas, will reduce food insecurity. In Zimbabwe's marginal lands, where climate change has led to increased frequency of extreme events, interest in maize has been rekindled (CADRI, 2017; Zinyengere et al., 2011). Given the roles of smallholder farmers in confronting the challenge of eradicating hunger and improving food security, farmers need to adapt to climate shocks. One way of adapting to climate-related shocks is by growing right maize varieties which are well suited to an environment and optimizing crop planting dates. There is a need to explore technologies like crop modelling on the concept of “more crop per drop”, if maize production is to increase (Waongo et al., 2015).

A Crop Simulation Model (CSM) is a mathematical model that describes crop growth and development processes as a function of weather conditions, soil conditions, and crop management (Raes, 2017). Models are tools which can be used to mimic reality (Keating and Thorburn, 2018). There are a wide range of crop growth simulation models, DSSAT CropSyst Jones et al. (2003), CROPWAT (FAO, 2018; Smith, 1992), CROPGRO (Boote et al., 1998), and APSIM (Probert et al., 1998). To address long-term climatic variability while avoiding costly long-term experiments, a common and well-accepted strategy is to combine results from short-term experiments with robust and validated dynamic crop models (Anar et al., 2019; Dzotsi et al., 2013). In the present study, we seek to use a hybrid approach to investigate the optimum planting dates for maize varieties. The CERES-Maize module's suitability in DSSAT was selected because the model has been successfully used across a broad range of soil, management, and climatic conditions in smallholder farming systems in southern Africa (Ngwira et al., 2014; Nyagumbo et al., 2017; Rao et al., 2015).

In Zimbabwe, crop models have been used to understand maize responses to environmental factors and management scenarios. They simulate process-level physiological responses of the plant to components of the soil–plant–atmosphere continuum (Chivenge et al., 2015; Zinyengere et al., 2015). To date, much of the researches into the variability of rainfall and maize varieties have focused much on monthly or seasonal rainfall totals and their relationship with land surface processes (Fiwa et al., 2014; Mhizha et al., 2014a; Vanuytrecht et al., 2014). When DSSAT is well-calibrated, the model can be a useful tool in enabling the development of water management strategies to improve crop production and water conservation, as well as to assess the impact of climate change on crop yields (Corbeels et al., 2016; Dzotsi et al., 2013; Jones et al., 2015). Dharmarathna et al. (2014) calibrated and validated DSSAT for planting dates in Sri Lanka. Wajid et al. (2014) calibrated DSSAT, modelling growth under varying nitrogen levels and planting dates on seed cotton.

In rain-fed production with erratic rainfall distribution, the date of the start of effective rain is a crucial factor in deciding when to plant and maize variety selection (FAO, 2013). Planting too early may lead to crop failure as critical growth stages may coincide with extended mid-season dry spells that have become a frequent occurrence (Hsiao et al., 2009; Mhizha et al., 2012; Vanuytrecht et al., 2014). On the other hand, planting too late may reduce the growing season and fully utilize growth resources. Both conditions can lead to yield reductions. Detecting when the effective rains start, the duration of the growing season and its end are essential for decision making (Keating et al., 2003; Mhizha et al., 2014a). The start date of the agricultural season is crucial as it determines sowing times and planting criteria. Also, this will aid in fitting adaptable varieties across different agro-ecologies. This type of crop production guidelines forms an important piece of information called a crop calendar.

A crop calendar is a tool that provides timely climate-related information about crops and varieties to promote local crop production (Steduto et al., 2009). It contains information on planting dates, sowing and harvesting periods of locally adapted crops in specific agro-ecological zones. These would go a long way in assisting smallholder farmers and extension officers. Previous attempts to study rainfall effects on crop yield were based on weather descriptors such as dates of onset and end of the rainy season, and temperature (Mhizha et al., 2014a; Mupangwa et al., 2011). Although these studies were comprehensive, farmers' practices and perceptions regarding start, duration and end of growing seasons are often not considered. Relatively fewer studies have investigated changes in intra-season rainfall characteristics, including, for example, the number of rain-days, frequency, and intensity of rain events in relation to crop varieties. Incorporating indigenous knowledge (farmers’ experiences) into climate change policies and crop production guidelines can lead to the development of effective mitigation and adaptation strategies that are cost-effective, participatory, and sustainable (Bekele et al., 2017). However, indigenous knowledge into climate change concerns should be used alongside scientific knowledge. Indigenous knowledge should complement, rather than compete with scientific knowledge.

Using a hybrid approach that combines CSM and Instat meteorological software to analyse past successful planting dates and use of indigenous knowledge helps develop maize cropping guidelines (Bekele et al., 2017). Such kind of climatic information is vital for smallholder farmers. It guides them in the choice of crops, varietal selection, planning of labour, on-time land preparations, when and how much moisture to trigger a planting event in rain-fed crop production (Waongo et al., 2015). The study aimed to use a hybrid approach to investigate the optimum planting dates for maize varieties and develop a maize crop calendar that would guide farmers on planting dates, dry spell probability during critical crop growth stages and rainfall cessation, using input from a survey of farmers as a basis of indigenous knowledge and cropping practices.

## 2 Materials and methods

### 2.1 The study area

**Figure 1:**
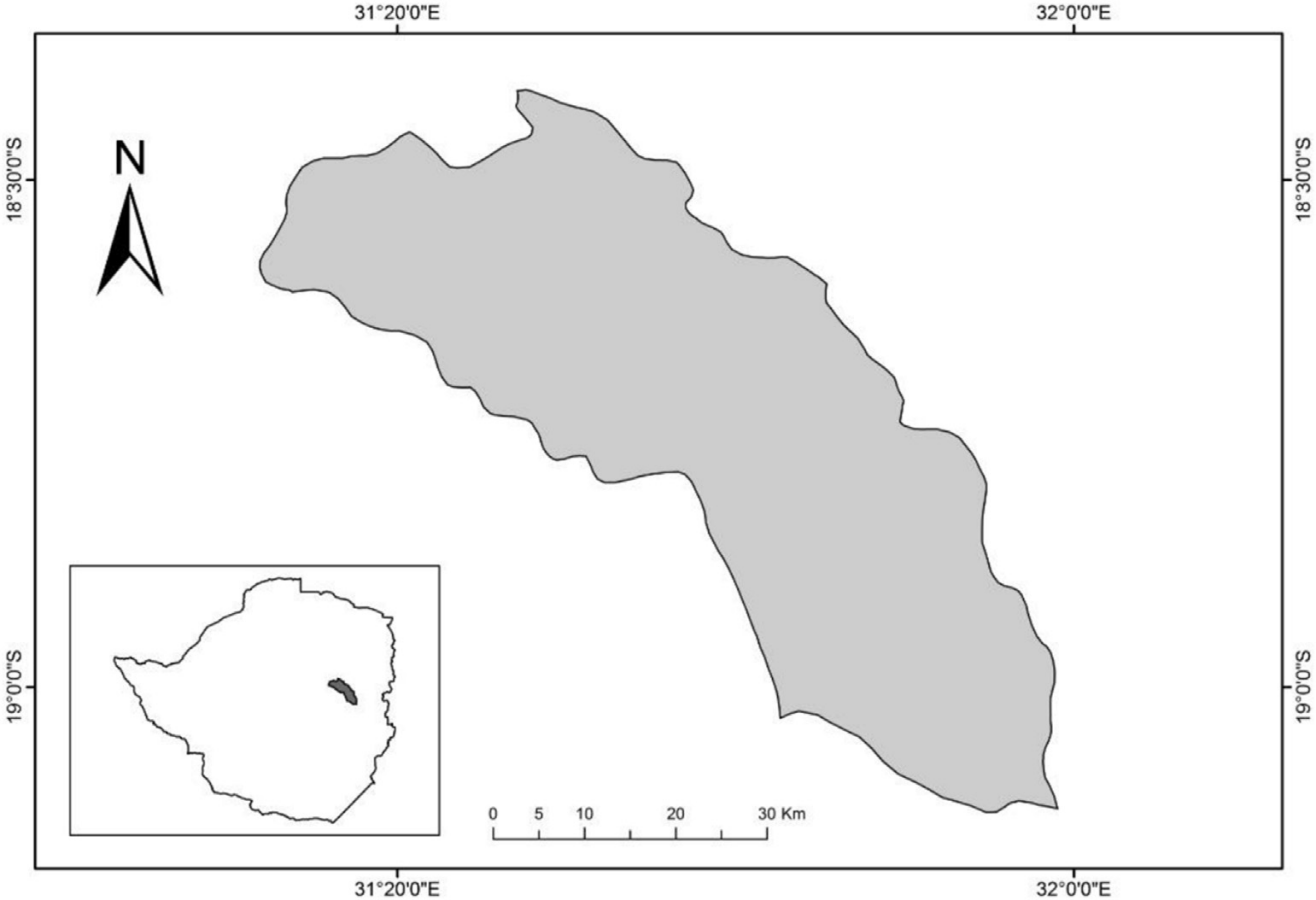
Map of Zimbabwe showing the location of Hwedza District in grey colour.

The study was carried out in Hwedza district of Zimbabwe (Figure 1). Hwedza climate station's geographic position is latitude -18°, longitude 31^o^ and altitude 1 425 m above sea level. The average total annual rainfall for Hwedza is 807 mm more than 90% of which is received in the rain season. The rainy season starts in November and ends in April. There is wide spatial and temporal variation in rainfall. Minimum and maximum temperatures are 11 °C in the winter months (June–July) and 33 °C in summer months. The mean annual temperature ranges from 18-19 °C. Livelihood strategies in Hwedza District are mainly cropping production-based, with crop-livestock systems focused on maize production and groundnut, soybean and cowpea. Figure 1 provides map of Zimbabwe showing the location of Hwedza District in grey colour.

### 2.2 Sampling design

A stratified two-stage random sampling design was used for the survey using a questionnaire. Stratified sampling its a probability sampling technique, where we divided the entire farmers into different farming sectors, then randomly selects the final farmers proportionally from the different sectors. In Zimbabwe, farmers are grouped into the following sectors: large scale commercial farmers, A2, A1, small-scale commercial sector, old resettlement and communal farmers. The farming sectors, also known as agricultural extension areas (AEA), constituted the strata. The sub-areas in each AEA formed the Primary Sampling Unit (PSU).

In contrast, farming households in the selected farming sector who grow at least one maize or sorghum field constituted the Secondary Sampling Unit (SSU). The sample size was based on the central limit theorem and sample size calculation formula based on the assumption of normal approximation (Majewsky et al., 2016). The sampling frame was 30 farmers per unit area/AEA; a census was used in areas where farmers were less than 30. The survey was carried out by government agricultural extension staff, and 438 farmers were interviewed through a structured questionnaire. Based on the assumption of normal approximation, more than 10% of farmers were interviewed per sector.

### 2.3 Season characteristics

The planting dates used for the study were based on the optimal plantings commonly used in Hwedza, Zimbabwe. In this study, Agriculture and Rural Extension (AREX) criterion currently known as Agricultural Technical and Extension Services (AGRITEX), Meteorological Services Department (MET) criterion and the Depth criterion (Raes et al., 2004) were utilised to determine planting dates from historical rainfall records. Amongst the three planting dates criteria, the Depth criterion was used to calibrate the DSSAT model. The common first sowing date derived from the survey was defined as the first rainfall event capable of supporting germination and establishment for 14 days).

The season was considered to have started if an area receives effective planting rains, as defined below. The INSTAT software was used to analyse events of interest (start of the season, dry spell, water balance) (Gallagher and Stern, 2015). Sowing dates were defined as:

1. Agriculture and Rural Extension **(**AREX) criterion in which sowing date is the first date from 1st October when an area receives more than 25 mm in 7 days with the condition that there is no 10-day dry spell or longer within the next 20 days (Mupangwa et al., 2011).
2. Meteorological Services Department (MET) criterion is the first date from 1st October when an area receives at least 40 mm in 15 days, with the condition that there is no 10-day dry spell or longer within the next 20 days (Mupangwa et al., 2011).
3. Depth criterion is the first date from 1st October when an area receives at least 40 mm in 4 days (Raes et al., 2004).

Numbers of rain days and temperature analysis: Descriptive statistics (minimum, maximum and range) were used to describe rainfall parameters and temperature. The following definitions were adopted from (Mhizha et al., 2014a; Raes et al., 2004):

• agricultural rain (wet) day: when an area receives 4.95 mm or more of rainfall
• rain day: when a region receives 2.95 mm or more of rainfall
• dry day: any day that accumulates less than 2.95 mm of rain
• waterlogging rains: At least 100 mm of rain within five consecutive days.

### 2.4 Model description

The Decision Support System for Agrotechnology Transfer (DSSAT) Version 4.7 is a computer software application program that encompasses crop simulation models for over 28 crops (Jones et al., 2003). The software DSSAT v4.7 requires databases for the weather, soil, crop management and experimental data (Liu et al., 2011). Crop models such as CERES-maize in DSSAT v4.7 can be used as decision support tools to determine best maize varieties selection and the optimum planting dates.

The CERES-maize model is a predictive, deterministic model designed to simulate maize growth, based on the soil water supply and crop water demand. A water stress factor decreases daily crop growth and consequently, maize grain yield to simulate the effects of limited soil water. The CERES-Maize model simulates maize yield under water limiting conditions by calculating potential evaporation; potential soil water evaporation and potential plant water transpiration derived from potential evaporation and leaf area index. The CERES-Maize model has been extensively used worldwide to simulate maize growth and grain yield and as a tool for planning and decision making by farmers in several countries (Corbeels et al., 2016). Comparison of simulated grain yield variety and observed data was done only for 2000–2008 due to the unavailability of longer-term maize actual yields data for the Hwedza district.

### 2.5 Model calibration

#### 2.5.1 Weather data

The four main weather parameters required for use by the DSSAT model are daily rainfall (mm), daily minimum (°C) and maximum temperature (°C) and daily solar radiation (MJ/m^−2^). Forty-nine years of daily rainfall, temperature and solar radiation data for Hwedza were obtained from the Meteorological Service Department (MSD) for 1949 to 2012. This period (over 30 years’ weather data) was chosen as acceptable by the World Meteorological Organization (WMO, 2012).

#### 2.5.2 Soil characteristics

Hwedza soils are classified as paraferralitic soils they range from felsic igneous and metamorphic rocks, moderately deep coarse loam (Mtambanengwe and Mapfumo, 2005). The DSSAT requires soil data for different soil profiles of specified depths. For each profile, the data required are the soil profile thickness, soil water content at field capacity (θ_FC_), at permanent wilting point (θ_PWP_), at saturation (θ_SAT_) and the saturated hydraulic conductivity (K_SAT_) (Vanuytrecht et al., 2014). Before the experiment, a soil profile pit of dimensions 1 m by 1 m by 1 m was opened, and disturbed soil samples were taken from 0-to 0.15, 0.15-to 0.30, 0.30-to 0.60 and 0.60- to 1.00 m depth to determine the soil physical characteristics or soil texture. The θ_FC_, θ_PWP,_ θ_SAT_ and K_SAT_ were estimated from soil texture and water characteristics equations using the Soil-Plant-Air-Water (SPAW) computer model. The soils are in order of Kaolinitic and group ranges from fersiallitic, paraferallitic and orthoferrallitic (Mtambanengwe and Mapfumo, 2005; Unversity of Zimbabwe, 1983) as in Table 1. S-build interface was used to input soil data for model calibration.

**Table 1:** Physical characteristics of the soils used in crop simulations (Nyamangara et al., 2000).

Depth (m)	0–15	0.15–0.30	0.30–0.60	0.60–1.00
Dry Matter %	99.7	99.2	98.8	98.2
Texture	mLS	mSal	cSaCl	cSaC
Gravel %	1	3	6	9
Clay %	4	18	29	39
Sand %	38	31	24	20
Exchangeable Ca (me %)	0.7	1.1	1.8	1.5
Exchangeable Mg (me %)	0.1	0.2	0.6	0.7
pH (CaCl_2_)	4.0	4.2	5.0	5.2
Exchangeable K (me %)	0.24	0.12	0.24	0.14
Total 'exchangeable bases-TEB (me %)	0.9	1.5	2.7	2.3
Cation exchange capacity (me %)	0.9	2.0	3.1	4.1
Base Sat %	100	75	87	57
Electrical conductivity (E/C)	21.3	11.4	10.6	10.6
Exchangeable potassium percentage (EKP)	25.6	6.1	8.0	3.5
Org Carbon %	0.29	0.19	0.14	0.17

#### 2.5.3 Crop management

In this study, three maize varieties and planting date criteria were optimised to find best varietal combination and planting date using the DSSAT model. The Crop Estimation through Resource and Environment Synthesis-CERES group of models requires parameters describing the crop environment interactions, termed genetic coefficients. The experimental data was collected by Zimbabwe's Department of Research and Specialist Services (DRSS) in demonstration plots in Hwedza. The collected parameters were phenology (flowering and physiological maturity) dates, seed maize yield and biomass at maturity, crop biomass and leaf area index (LAI) at different stages of growth, rooting depth and soil profile distribution, and soil water content measurements. To use the CERES maize models in Zimbabwe, the genetic coefficients of the widely grown varieties were obtained from the International Maize and Wheat Improvement Centre (CIMMYT) (Jones et al., 2003) (Table 2). The calibrated crop files and crop varieties are described in Table 2. The only difference between the three varieties was their length of the growing cycle and their time to reach various phonological stages. Therefore, three files were created short, medium and long maturing varieties. The maize genotypic input parameters within the CERES maize module were marginally adjusted within a ±5% margin to mimic observed average district grain yield.

**Table 2:** Genetic coefficients of Zimbabwe maize varieties.

Maize variety	Days to flowering	Days to maturity	P1	P2	P5	G2	G3	PHINT
Short	45–50	90–110	110	0.3	680	820	6.6	38.9
Medium	56–65	111–130	200	0.3	800	700	8.5	38.9
Long	66–75	131–155	320	0.52	940	620	6	38.9

P1: length of the juvenile period (degree days above 8 °C.

P2: Factor to account for the delay in development when day length is less than the optimum.

P5: Time in degree days from silking to maturity.

G2: Maximum kernels per plant.

G3: Kernel filling rate (mg/day) during grain filling under optimum conditions.

PHINT: Phyllochron interval (time in degree days between successive leaf tip appearance).

##### 2.5.3.1 Crop parameters

A planting density of 37 000 plants/ha was used; the average plant population was based on farmers’ practices. Two levels of fertilizer application were used:1) an optimal' level of67 kg N/ha, 30 kg P_2_O_5_/ha and 11 kg Ca/ha; and 2) less than optimal 33 kg N/ha, 15 kg P_2_O_5_/ha and 6 kg Ca/ha. This is representative of resource-poor farming communities in Hwedza. The maize yield adjustment was less than optimal of 33 kg N/ha, 15 kg P_2_O_5_/ha, and 6 kg Ca/ha fertilizer application rate was used.

### 2.6 Model validation

Validation of model performance was done using observed data. The data was obtained from Hwedza district from 2000 to 2008. The medium season variety was used to validate simulated results because several farmers indicated that they prefer to plant medium-season varieties. The model was used to capture seasonal mean crop yields and temporal yield variation in response to climate. There was a challenge of using one station data to simulate maize yields of smallholder farmers. Generally, farmers operate under varying conditions, even in the same district. Therefore, reported district-wide average yields reflect varying conditions like climate, soils, crop varieties, sowing time, planting densities and fertilizer application.

The simulated yields would be expected to be considerably different from averaged district yields for yield bias correction. The simulated yields at district level were corrected against the observed district yields for a more representative assessment of model performance. Ideally, an extensive series of district yield data and a detailed description of the variation in physical and management conditions over space and time would compute bias satisfactorily. Model performance was evaluated by comparing simulated and measured values of maize grain yields for 7 years. During calibration 2006 was removed because of unavailability of climatic data. Planting dates for yield simulation were derived from scenario analysis from historical rainfall data using INSTAT.

### 2.7 Data analysis and evaluation of the model

Survey and modelling data were analysed using descriptive statistics in Statistical Package for the Social Sciences (SPSS) (Chandler, 2010). The statistical package generates descriptive statistics on the start of the season, end of the season, mid-season drought periods, and finding major cultivated varieties in Hwedza. For model calibration, evaluation, and improvement, crop and soil measurements are required to make comparisons between simulated and observed data Hoogenboom et al., 2019. Crop model performance was evaluated using the root mean square error (RMSE) (Hoogenboom et al., 2019). This study evaluated the performance using test data limited to yield only. The accuracy of DSSAT was assessed using the root mean square error (RMSE) Eq. (1) (Yang et al., 2014)

(1) $$RMSE={\left[\frac{\sum_{i=1}^{n}{\left(S_{i}-O_{i}\right)}^{2}}{n}\right]}^{1/2}$$

where: S_i_ = measured value, O_i_ = estimated value of y and n = number of observations.

The target value for RMSE is 0, and RMSE values close to 0 indicate a good agreement between observed and simulated data. RMSE indicates the deviation of the simulated values from the measured values with estimates approaching zero, indicating the better performance of the model and good agreement of the measured to the simulated values. The post hoc tests at (alpha = 0.05) were used to confirm where the differences occurred between planting criterion and maize variety (Affholder et al., 2013).

## 3 Results

### 3.1 Farmers experience and historical climate data characterisation

**Figure 2:**
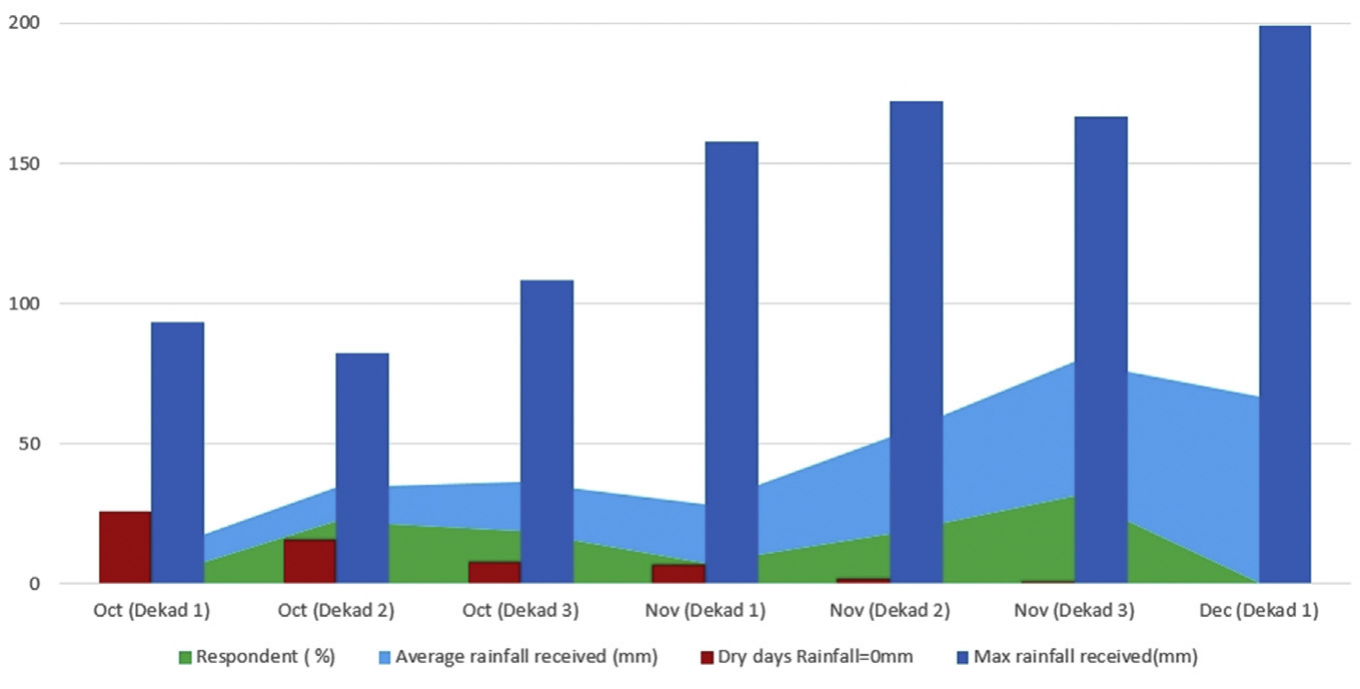
Start of a season based on farmers' experience and historical climate data in Hwedza district.

Figure 2 presents a summary of farmers' indigenous knowledge on the start of the season. Survey results showed spread variation concerning the start of the season and depending on geographic areas. There was no consistency concerning what farmers reported to be the date for the start of the season. Farmers respondents were very variable across the month but with a systematic pattern where the least responses were always on each month's 1st dekad. Farmers responded to rainfall availability to kick start planting activities (Figure 2). There is an inverse relationship between maximum rainfall received and the number of dry days from 1^st^ dekad of October to 1^st^ dekad of December. The highest number of farmers (31.4 %) indicated that they preferred to plant on the 3^rd^ dekad of November, which corresponds with 48.1 mm of rainfall. There are fewer chances of not receiving rainfall on 3^rd^ dekad of November each year and the lowest number of farmers (0.2%) planted on the 1^st^ dekad of December (Figure 2).

### 3.2 Characterisation of Hwedza station historical climate data

**Figure 3:**
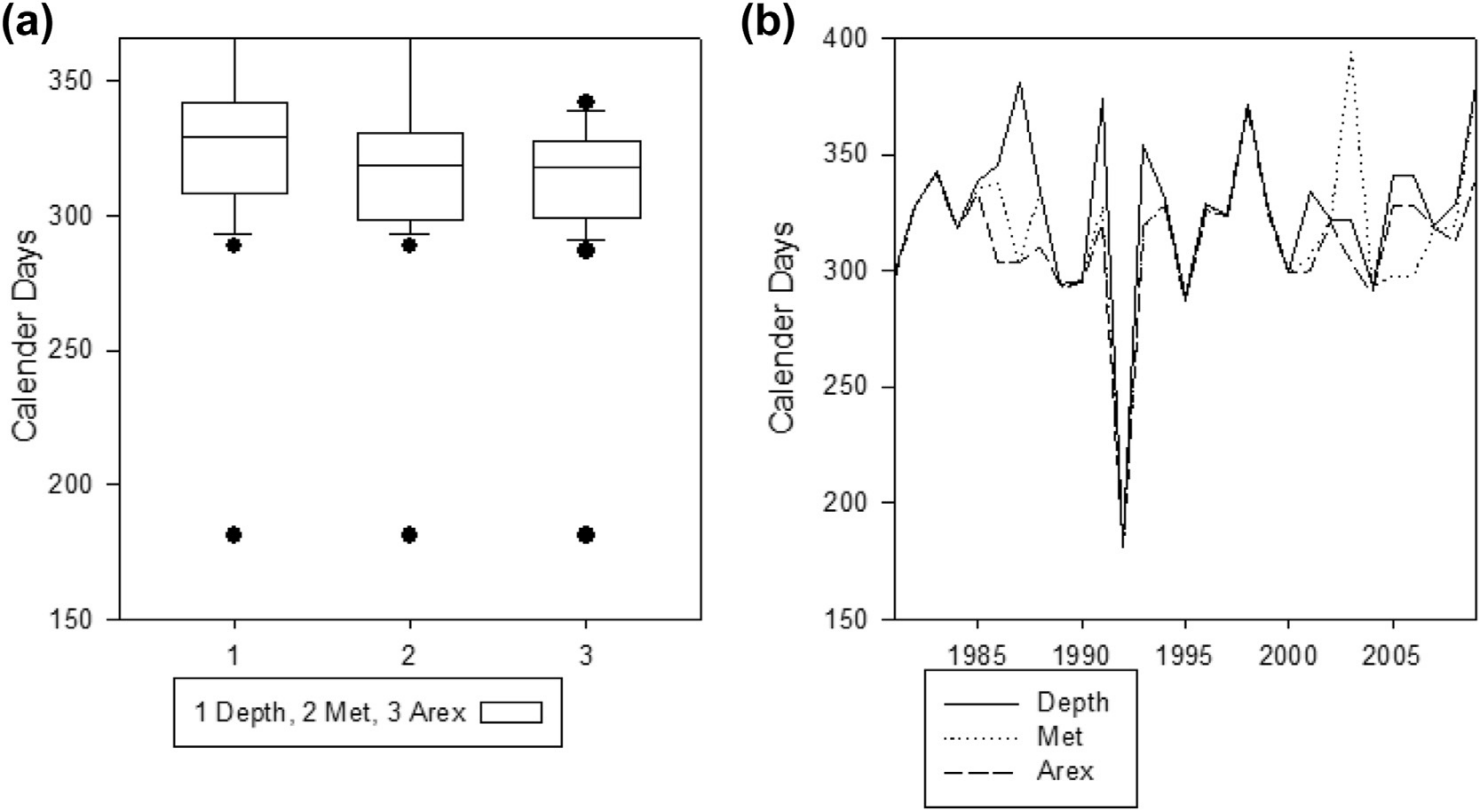
(a) Vertical boxplot showing criteria used to determine the start of the season. (Days numbers were counted starting from 1 January) (b) Trend analysis for three planting criteria (1-Agriculture and Rural Extension**-**AREX, 2-Meteorology-MET,3- Depth criterion) from 1980-2010.

Figure 3 presents the start of the agricultural season using historical climate data for 30 years. Three criteria for determining the start of the season were analysed using INSTAT (Raes et al., 2004). According to the AREX criterion, the season started on the 2^nd^ dekad of November (11 November), while the MET criterion the season usually started on the 2^nd^ dekad of November (16 November). There was no start of the season in 1992 in all three criteria; the results are shown by the minimum value of day calendar (30 June-181). The Depth criterion gave a common start of the season on the 1^st^ dekad of December, the 3^rd^ of December. The *F*-tests of correlations (p = 0.05) (Figure 3) indicated no significant trend in the planting dates for any of the three criteria over the past 30 years. Planting threshold for AREX was first to be met, followed by MET criterion and lastly Depth criterion. There was more variability in Depth criterion (Figure 3).

**Figure 4:**
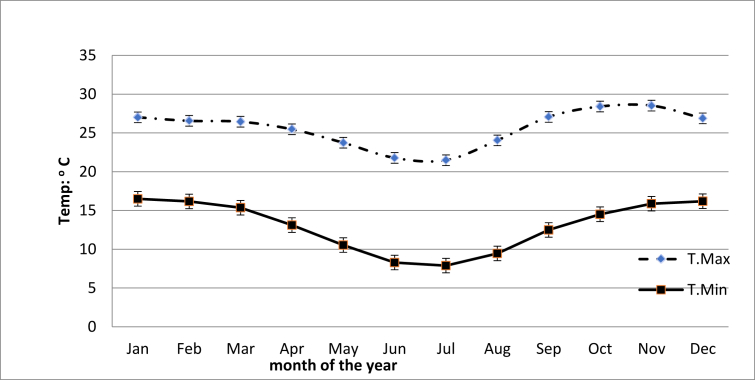
Monthly mean maximum and minimum temperature (dashed line = T_max_; continuous line = T_min_).

Figure 4 shows the average monthly temperature Hwedza station. Lowest minimum and maximum temperature are recorded in July 7.8 °C and 21.4 °C, respectively. The highest maximum temperature recorded in November 28.5 °C and highest minimum temperature January 16.5 °C.

**Figure 5:**
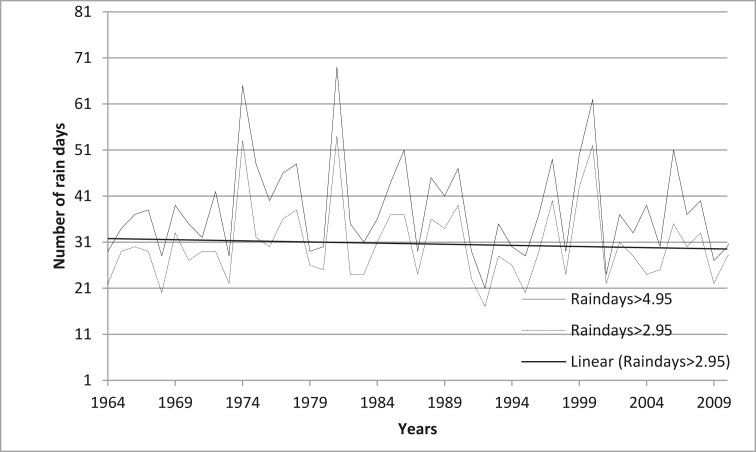
The number of rain days in a season at Hwedza Meteorological Station in Zimbabwe (dashed line = rain days> 2.95; continuous line = rain days> 4.95 mm).

Figure 5 present number of rain days of Hwedza station for 1964–2012 to understand the change rainy days. The value R^2^ (0.0130) for 4.95 mm and R^2^ (0.073) for 2.95 mm rain-days indicate that from 1949-2012 there is less or non-variability of several rain days around its mean. Comparing 4.95 rain day is becoming more variable R^2^ (0.0130) than 2.95 mm rain-day R^2^ (0.073). The lowest rain days were recorded in 1992, which correspond with no start of the season and then the lowest simulated yield in 1992 (Figure 5).

**Figure 6:**
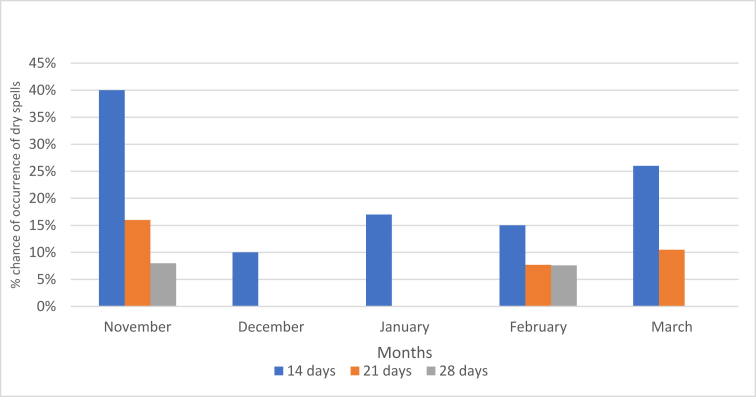
Risk of 14, 21- and 28-days’ dry spells during the growing season at Hwedza station.

Figure 6 depicts the chances of having dry spells of 14, 21, and 28 days. The percentage chance of having dry spells >14, 21, and 28 days is higher during the early parts of the growing season, which continues to decrease to the peak of the rainy season and increases further towards the end of the season. The risk of having 14–21, and 28-day dry spells during the growing season at Hwedza Meteorological station is presented (Figure 6). The dry day was defined when rain recorded in rain gauge was less than 4.95 mm per day.

### 3.3 Modelling results

**Figure 7:**
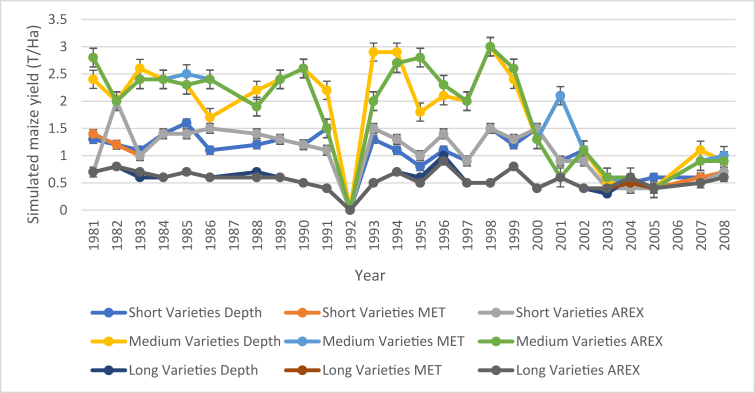
Effect of planting criteria on simulated average yields in (t/ha) from 1981-2008.

Figure 7 present simulated maize yield results (1981–2008) to identify the best sowing options (variety and sowing date) from different planting criteria. The simulated yield ranges 0–2.8 t/ha. The lowest yields (0 t/ha) across all planting criteria and maize variety were simulated in 1992, corresponding to no start of effective rains on a drought season (Figure 7) and lowest rain days (Figure 7). The highest yields (2.8 t/ha) were simulated from both AREX and MET criteria but from a medium variety in 1981 and 1995 (Figure 7).

**Figure 8:**
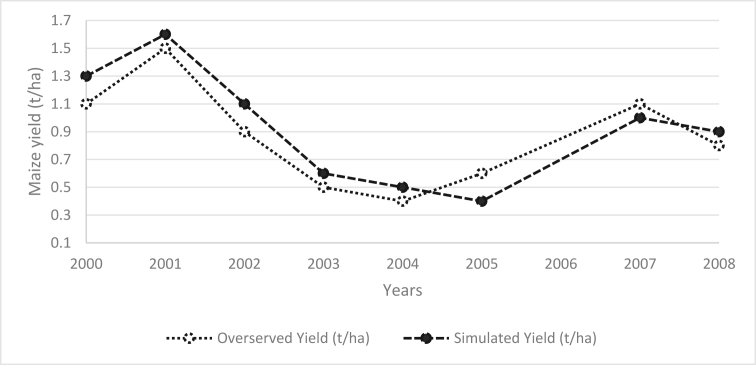
Hwedza district, Zimbabwe observed and simulated maize yields from 2000 to 2008.

Local calibration and validation were successfully carried out for yield simulation. Model performance was good (RMSE-0.20). The model underestimates maize yield by a mean deviation of -0.06 t/ha and slightly overestimated in 2005 and 2007 (Figure 8). There was a positive correlation (R^2^ = 0.88) between observed and simulated yield.

**Figure 9:**
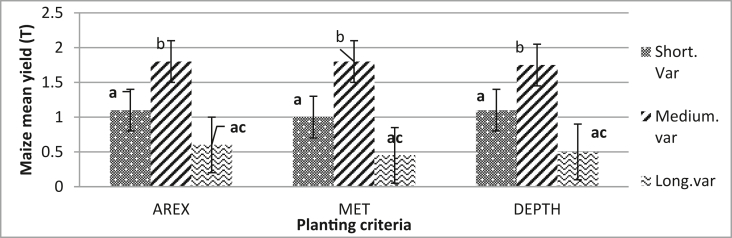
Identification of the best sowing option (AREX, MET, DEPTH criterion, and best maize yield at Hwedza district. Bars with different small letters denote significant difference in treatments from their respective control at P > 0.05, analysed by Dunken post hoc test.

The best sowing option (variety and sowing date) for maximum mean maize yield is presented in Figure 9. In all combinations of planting criteria and maize varieties, the standard deviation error bars did not overlap; it is a clue that the difference may be a significant and statistical test to conclude (Table 3). Table 3 presents best yielding maize varieties (medium-term variety P = 0.663, Shor term variety P = 0.169 and long maize variety P = 0.004). The post hoc tests (alpha = 0.05) confirm the differences between medium and short varieties. Also, there is a significant difference between medium and long varieties, whilst there is no significant difference between short and long varieties.

**Table 3:** Identification of best maize yield varieties for Hwedza District.

Sample	Mean (T/Ha)	Variance	Standard deviation	Standard error of mean	P. Value
Baseline yields	1.022	0.168	0.410	0.137	
Medium term variety	0.925	0.169	0.412	0.146	0.633
Short term variety	0.750	0.130	0.361	0.128	0.169
Long variety	0.479	0.009	0.099	0.035	0.004

Medium-term variety simulated the highest yield 0.925 t/ha followed by a short variety of 0.750 t/ha and long-term variety simulated at 0.479 t/ha. Mean separation was used to determine which varieties are most suitable in Hwedza and were compared with observed maize district average yields (Table 3). The simulated results for the medium season and short-season varieties showed no significant difference from the observed maize district average yield, P = 0.6 and P = 0.16. In contrast, yield difference was obtained between simulated results for long season varieties and district average, P = 0.004. Identification of the best varieties for maximum mean yield is shown in Figure 9; medium maize variety indicated the highest mean yield from 2000 to 2008, followed by short varieties and lowest yields were recorded on long maize variety.

Relationship between total seasonal rainfall, simulated yield and observed yields.

### 3.4 Crop calendar for maize

**Figure 10:**
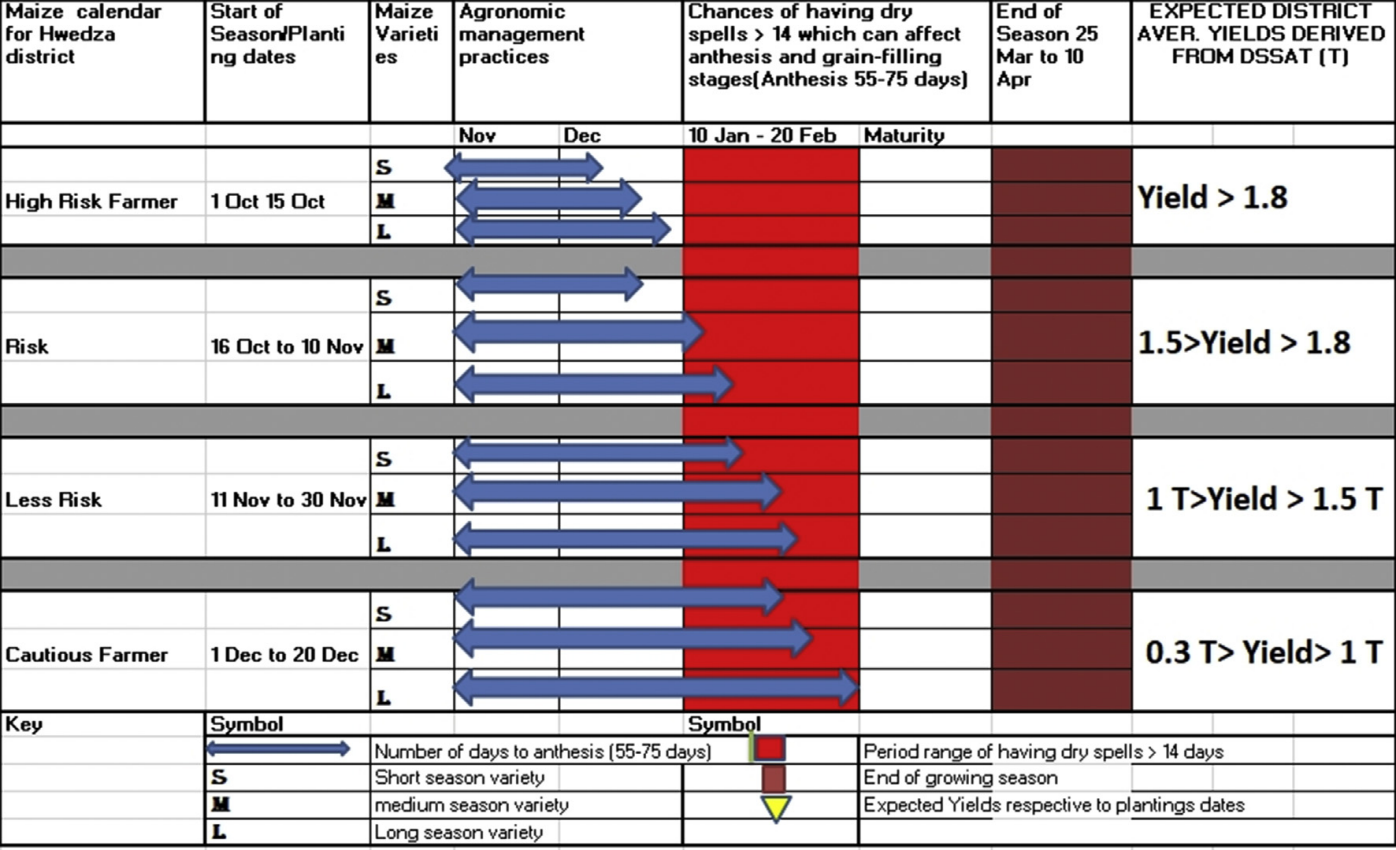
Maize crop calendar for Hwedza District.

The decision support tool for maize production was developed for Hwedza district (Figure 10). The tool was developed based on triangulation of the survey results, analysis of 49 years’ historical climatic data for Hwedza climate station and simulation results by the DSSAT model. The tool is meant to allow maize producers to make decisions for maize production management based on climate variability in Hwedza. The decision support tool indicated that when they plant maize early, yields as much as 1.8 t/ha can be attained if the long, medium and short varieties are planted between 1 October to 10 November using proper fertilizer applications.

The highest maize yields in the crop calendar are obtained with October plantings due to favourable heat units for hybrid maize growth during the most critical stages of growth (anthesis and kernel development) (Mhizha et al, 2012, 2014a; Zinyengere et al., 2014), but growth for this planting time can usually only be sustained with irrigation. However, plantings in October may be feasible with early rains (over 40 mm), and early planting in October in Figure 10 has a lower chance of successful crop establishment due to drought periods which can often occur soon after early planting (Figure 10). Moreover, planting in December can also produce low yields and is very risky because the crop may suffer from water stress at the end of the season; pests like stalk borer and (maize streak virus) are more prevalent late-planted maize crops.

The use of the DSSAT model, the length and the quality of the growing period can be assessed for its suitability to the maize varieties (Chisanga et al., 2017; Chivenge et al., 2015; Ndawayo et al., 2017; Zinyengere et al., 2015). This research developed a crop calendar that can indicate whether to grow maize varieties of a particular length of the growth cycle for Hwedza district. It contains information on planting, sowing and harvesting periods of locally adapted crops in specific agro-ecological zones. Crop calendars also provide information on the sowing rates of seed and planting material and the main agricultural practices (Van Der Velde et al., 2012). It is a tool developed to assist farmers, extension workers, civil society and the private sector to access and make available quality seeds of specific crop varieties for a particular agro-ecological zone at the appropriate sowing/planting season. Development-aid workers can use it to plan and implement seed relief and rehabilitation activities following natural or human-led disasters. Furthermore, the crop calendar can serve as a quick reference tool in selecting crop varieties to adapt to changing weather patterns accelerated by climate change.

## 4 Discussion

### 4.1 Farmer perception on the start of the rainfall

Farmer's indigenous knowledge and experiences are essential in farming systems. It is crucial to understand how farmers respond and adapt to ecophysiology. Understanding farmer's indigenous knowledge, especially on how farmers' agronomic production practices respond to climate. This kind of participatory approach marks the starting point of scientific research (Hazer Sancar, 1993; RÃ-os-Carmenado et al., 2012).

The study results show a higher proportion of female-headed farming households in Hwedza compared to a national female household headed at a national level, which stands at 21%. Women play a vital role in the rural agriculture economy, especially in developing countries. Improving woman access to the land, credit, inputs agricultural training and information compared to men has been shown to significantly increase productivity, reduce hunger and malnutrition and improve rural livelihoods (Doss et al., 2011). The survey data showed that all farming households in Hwedza grew maize, while only 8.7% grew sorghum. This is because maize is the main staple food crop in Zimbabwe and several government initiatives support its production.

### 4.2 Characteristics of Hwedza climate

Indigenous knowledge provides the basis for problem-solving strategies for local communities, especially in a farming system where 80% rely on rain-fed production. Results from indigenous and scientific knowledge systems need to be triangulated to form best adaptation strategies like crop calendars (Nidumolu et al., 2015). There was no consistency concerning what farmers reported to be the date for the start of the season. This might be attributed to farmers’ different perception of the start of the season (Zinyengere et al., 2015).

The results from both survey and analysis of historical climatic data indicated that having effective rains in the 1st dekad of October was low, as shown by a high frequency of days with no rainfall. This was consistent with survey data that showed only 2.10% of farmers indicating that sometimes the season starts as early as 1^st^ dekad of October (Figure 2)). Generally, smallholder farmers start to do land preparation after receiving first rains. Smallholder farmers are not usually risk-takers; there is a lagging period or response time between rainfall and the real planting activity. The delay culture by farmers not to promptly plant with effective rains might be due to poor planning, failing to procure agriculture inputs in time, late land preparation, and fear of the false start of seasons. False start of the season causes poor crop establishment causing the farmer to do multiple re-plantings. In Zimbabwe's sub arid and arid environment, rainfall above 25 mm can support maize seed emergence, germination, and establishment for 14 days (Mhizha et al., 2014b; Raes et al., 2004).

Planting with first effective rains improves the maize crop's chances to utilize heat units for robust growth, and the crop can use the length of the growing period. Therefore, farmers wait for cumulative rain to make soil to reach field capacity. There are high chances of having successful maize planting as season progress to 2^nd^ dekad of November to December as indicated by results from both survey from which 31.4% planted in the last dekad of November and historical climatic data (Figure 2).

#### 4.2.1 Analysed start of the season from historical data using INSTAT

The variability of onset dates further proved that rainfall pattern and distribution from one station could not provide operational guidelines for the entire district (Figure 2). Soils need to have enough moisture to support germination and emergence for 10–14 days depending on soil types and prevailing weather conditions. Three different criteria (AREX, MET, and Depth) to determine the season's start were analysed. Analysis of planting criteria Raes et al. (2004) the DEPTH criterion is more difficult to meet because it requires the highest moisture to bring dry topsoil at a wilting point to field capacity and support seed emergence until the next rains are received. First, effective rains mark the start of the planting of crops. The earlier crops planted, the higher the probability of having higher yields because the crop will utilise heat units in October, November and December. Boxplot (Figure 3) indicates there is no significant variation between AREX and MET criterion. Though the AREX and MET criterion had the earliest dates of planting, they also have a big chance of having dry spells more than 14 + days in October and November (Figure 6). Planting with earliest rains in rain-fed maize production has high chances of replanting's (Mhizha et al., 2014b). The start of season analysis might not represent the whole study area because only one station was used to characterise the climate data. This is a primary limitation to Zimbabwe and other regional states where meteorological stations have been declining over the years. In this regard, the use of climate space data should be explored to provide spatial and temporal meteorological data in areas where there are no meteorological stations (Michaelides et al., 2009).

#### 4.2.2 The occurrence of dry spells

The risk of 14, 21 and 28-day dry spells during the growing season at Hwedza Meteorological station is presented in Figure 6. Dry spells cause intermittent water stress in maize. Water is one of the primary yield-reducing factors in maize production. Availability of adequate soil water in the root-zone is essential for proper maize development. Moisture stress occurring at critical growth stages (anthesis and grain filling) of maize causes yield losses (Basir et al., 2018). Potential yield can be reduced by 10–50% if moisture stress occurs at 8–16 leaf stage of maize phenology. During this period, water stress will reduce ear size and potential yield (bib_citation_to_be_resolvedUÃ§ak et al., 2014).

There was a 17% and 15% chance of drought periods exceeding 14 days in January and February (Figure 6, respectively) which would most likely reduce maize yield. In Hwedza, rain-fed maize reaches anthesis between January and February. The critical maize growth stages (silking and grain filling) often coincide with mid-season dry-spells. The silking stage is the most sensitive stage for water stress. High temperatures and water stress during silking can result in 100% loss (Killi et al., 2017; Neiff et al., 2016). This explains the importance of growing varieties that match the growing period's length with sufficient soil water availability. Dry weather that starts early and extends over several growth periods (terminal stress) will have a compounding effect with severe reductions in maize and sorghum yields. False starts to the rainy season will cause replanting and increase the cost of rain-fed crops production; hence the start of the season needs to be monitored to reduce crop failure risk (Mupangwa et al., 2011).

### 4.3 Model performance and output

In crop management, several factors may affect choosing the best or optimum planting date and choice of maize varieties. Start of effective rains in an agricultural season is one of the main concerns because it determines the amount of water available to establish the crop, especially in water-limited areas or drought-prone regions. Simulated maize yields explain the DSSAT model's ability to mimic specific processes happening in the farming system (Jones et al., 2003). The deviations between averaged simulated and measured annual maize grain yields were small for 2004 (residual = -0.06 kg ha^−1^) (Figure 7). There were some systematic deviations in the model simulations, and that the 8-year average annual yield was simulated reasonably well. The root means square error (RMSE) (0.20) (Figure 7), which is an overall measure of model performance. This is probably because genetic coefficients were obtained from a limited set of field observations. The genetic coefficients were obtained from 3 major seed houses. Using generalised genetic coefficients (the kernel weight G1 and kernel number G2) may explain why simulated grain yields were not in good agreement with the observed values (Figure 7). The Crop Simulation Model-CSM-CERES-Maize model uses six variety coefficients, three representing early growth (P1, P2 and P5), two representing grain filling (G2 and G3), and one representing the phyllochron interval between successive leaf tip appearances (PHINT) (Lizaso et al., 2008). Cultivar coefficients must be calibrated to meet the observed yield or biomass under no stress growing conditions, without water, heat or nutrient deficiencies (Jones et al., 2003; Liu et al., 2011).

The model was successfully used to investigate the relationships between crop management practices (such as cultivar selection and planting date), and the environmental factors (essentially soil properties and weather conditions), which interplay ultimately determining final crop yields (Hoogenboom, 2000; Jones et al., 2003). Maize yield was affected by planting date, and in our simulations, there was no yield for the year 1992 (Figure 6). There was no start of season and year was characterized by very long drought periods. The simulated results showed that DSSAT is sensitive to moisture stress. However, it failed to simulate 1992 yields because of the drought experienced in that season. In years where total seasonal rainfall was greater than 700 mm, simulated, and observed yields were greater than 1 t/ha. All years with total seasonal rainfall less than 700 mm did not have yields, and this proves that rainfall distribution (rain days) is more important than seasonal totals. Lowest maize was from long term maize variety because long term varieties have longest anthesis and grain filling period which can be interrupted by lack of moisture in rain-fed maize production. Water deficit may develop any time during the life cycle of the crop, affecting T_r_ and hence biomass accumulation, depending on timing, severity, and duration of the stress (Corbeels et al., 2016; Mhizha, 2010).

#### 4.3.1 Evaluating planting criteria and maize varieties

The variance ratio for planting criteria to maize varieties did not differ, as indicated by standard mean errors bars (Figure 8). The combination of AREX criterion and maize varieties produced the highest yields followed by the MET criterion and the Depth criterion. The results show fewer differences in yield obtained from simulation with a different combination of planting criteria. The combination of Depth criterion and medium season maize variety is recommended because depth criterion has less risk of crop failure (Raes et al., 2004). By planting late, farmers avoid the potential false starts which may occur early in the season. This ensures that crops do not suffer moisture stress in the initial and crucial development stages (Chivenge et al., 2015). In rain-fed maize production, the maize yield of short and medium variety was not much affected by the time of planting (Figure 8).

Medium and short varieties (P = 0.6333 and P = 0.169) respectively, showed no difference between the means, and we conclude that a significant difference does not exist. The results indicated that medium-term varieties (0.925 t/ha) are the most suitable maize variety in Hwedza, followed by short term varieties (0.750 t/ha) and lastly long term (0.479) varieties (Figure 9 and Table 3). The results provided in Table 3 are consistent with the observation by Mhizha et al. (2014a) and Phillips et al. (2006) that medium-term varieties are dominant in agro-ecological region IIb and III in Zimbabwe, while in drier zones the more drought-tolerant crops, pearl millet (*Pennisetum glaucum* L.) and sorghum (*Sorghum bicolour* L.) are recommended. Some farmers are risk-takers and grow long term maize varieties (P-value < 0.05) (Table 3) our results showed that there's a difference between the means of long-term varieties. Climate variability confuses farmers so that they choose maize varieties which don't match with crop water requirements. In some years' farmers do benefit from well-distributed rains which favours better yields from long-term maize varieties.

#### 4.3.2 Relationship between total seasonal rainfall, simulated yield and observed yields

The correlation p = 0.0049 between rainfall characteristics and maize yield shows that an annual number of the rain days had a strong (significant) positive correlation with maize yield. In contrast, dates of onset (r = 0.003) had weak correlations with maize yield. This implies that variations in annual rain days and annual rainfall amounts account for most annual variations in maize yield. This confirms the findings by (Mhizha et al, 2012, 2014a; Raes et al., 2004; Zinyengere et al., 2014) that annual rain days and the annual amount had the most significant effect on maize yield. Thus, the higher the amount of rainfall spread over the number of rain days in a year, the greater the maize yields.

Severe mid-season drought periods greater than 14 days occur at anthesis and grain filling stages, and final maize yields are affected. Maize requires about 600 mm of even rainfall distribution to reach physiological maturity. Growing small grains is highly recommended in low seasonal total rainfall (less than 600 mm) and few rain days (uneven rainfall distribution). In Hwedza, only 7.8 % of farmers grew sorghum (or other small grains) which implies that farmers are advised to grow both maize and sorghum to reduce the negative impact of climate variability on yields (Chimonyo et al., 2016).

#### 4.3.3 Limitations

A hybrid approach is a practical and applicable method to determine best planting windows and maize varieties in Hwedza in Zimbabwe. However, the exercise of using a crop model in a maize production environment needs to consider farmers experience, soil scientists, pest discipline scientists and economists to come up with guidelines that are evidence-based to farmers (Ahmad et al., 2020; Boote et al., 2018; Liu et al., 2020). The proposed maize guidelines are designed for site-specific simulations because they use one-point weather station, which might not represent the entire district. The study used historical climate data; prevailing management practices, typical soil characteristics were used to develop planting dates for maize production but climate change, the model may need to be re-run with up to date climatic data, future climate scenarios to come up with updated and relevant guidelines for future use (Boote et al., 2018).

The developed sowing guidelines were presented in a crop calendar (Liu et al., 2020; Mhizha et al., 2014a). However, the set guidelines are still rather general, and their application at the farm level is therefore difficult unless they are simplified to farm level. Thus, the guidelines proposed have a limitation in spatial simulation (Hoogenboom, 2017). The hybrid method used has many current and potential uses for answering questions in research, crop management, and policy. However, the model uses’ cautions and limitations depend on whether the model complexity is appropriate to the question being asked and whether the model has been tested in diverse environments (Hoogenboom et al., 2019; Jones et al., 2015). Also, the method needs to consider, long to short-term weather forecasts. Use of weather forecasts could help farmers know when the likelihood of having dry spells, wet spells and other extreme events to improve on-farm operational activities (Waongo, 2015).

## 5 Conclusions

The maize cropping calendar (crop management guidelines) was developed as a general guide applicable to the study area. The cropping calendar indicated that medium and short-term varieties (P = 0.6333 and P = 0.169) respectively showed no maize yield difference.

The survey helped to find agro-meteorological challenges which are affecting maize production in Hwedza district of Zimbabwe. There is high variability in rainfall characteristics (start of the season, number of rain days and drought length) which translates to high variability in maize yield per hectare. The result also reveals that the number of rain days is the strongest influence on maize yield per hectare in the study area. The validated DSSAT model and INSTAT were used to select the best combination of planting criteria and maize variety. Crop simulation model (DSSAT) integrates various processes in the soil-crop-atmosphere continuum that determine crop growth and production. The study showed that maize variety and planting date affect maize yields considerably. The AREX criterion and medium season variety produced the highest district average yield of 0.9 t/ha. The methodology used can be used to select potential maize varieties that can be grown in an area before establishing costly field experiments and can also determine the optimum planting dates. Hence, DSSAT is a useful tool to investigate management strategies to optimize crop productivity. Regarding the findings of planting dates and maize varieties based on the approach, the cropping guidelines have to be associated with other management strategies to strengthen climate change adaptation.

## Declarations

### Author contribution statement

Hillary Mugiyo: Conceived and designed the experiments; Performed the experiments; Analyzed and interpreted the data; Contributed reagents, materials, analysis tools or data; Wrote the paper.

Teddious Mhizha: Conceived and designed the experiments; Performed the experiments; Wrote the paper.

Vimbayi. G.P. Chimonyo: Contributed reagents, materials, analysis tools or data; Wrote the paper.

Tafadzwanashe Mabhaudhi: Analyzed and interpreted the data; Wrote the paper.

### Funding statement

This research did not receive any specific grant from funding agencies in the public, commercial, or not-for-profit sectors.

### Data availability statement

Data included in article/supplementary material/referenced in article.

### Declaration of interests statement

The authors declare no conflict of interest.

### Additional information

No additional information is available for this paper.

## Appendix A Supplementary data

Maize and Sorghum Questionnaire Maize and Sorghum Questionnaire
